# Fluoride therapy in the prevention of dental caries

**DOI:** 10.1186/1824-7288-40-S1-A71

**Published:** 2014-08-11

**Authors:** Laura Strohmenger

**Affiliations:** 1 Department Health’s Science, H.San Paolo, Milan, Italy

## 

The document "National Guidelines for the prevention and oral health promotion in childhood", 2013, is an act of address for those involved in the management of oral health and in particular to the pediatrician, because the figure of the pediatrician in the prevention of oral health is absolutely critical, as confirmed by the entire international literature in recent years.

Correct attitudes and behaviors adopted since childhood that will allow the child to protect his health.

The fluoride is the cornerstone of prevention of tooth decay and is required for all individuals.

Over the years, have been developed different means of administration of fluorine, each with different strengths, dosages and frequency of use [1-5].

Fluoride supplements should be prescribed by the pediatrician in cases of real difficulty for topical administration of fluoride through toothpaste or fluoride added as a method of in subjects at risk of tooth decay [6].

The decline of caries in our country it is highly likely also due to the pediatrician who, using the national guidelines, can inform parents and families induce the acquisition of preventive behaviors currently defined by scientific research [7].

To this end, again, the ministerial guidelines on the subject that were reviewed by a team of experts representative of the Italian research in this area.

## References

[B1] Marinho VC Higgins JP Logan S Sheiham A Fluoride gels for preventing dental caries in children and adolescents Cochrane Database Syst Rev 2009 2 CD002280 12076446 10.1002/14651858.CD002280

[B2] Marinho VC Higgins JP Logan S Sheiham A Fluoride mouthrinses for preventing dental caries in children and adolescents Cochrane Database Syst Rev 2009 3 CD002284 12917928 10.1002/14651858.CD002284

[B3] Marinho VC Higgins JP Logan S Sheiham A Fluoride varnishes for preventing dental caries in children and adolescents Cochrane Database Syst Rev 2009 3 CD002279 12137653 10.1002/14651858.CD002279

[B4] Marinho VC Higgins JP Sheiham A Logan S Combinations of topical fluoride (toothpastes, mouthrinses, gels, varnishes) versus single topical fluoride for preventing dental caries in children and adolescents Cochrane Database Syst Rev 2009 1 CD002781 14973992 10.1002/14651858.CD002781.pub2 PMC6999808

[B5] Marinho VC Cochrane reviews of randomized trials of fluoride therapies for preventing dental caries Eur Arch Paediatr Dent 2009 10 183 91 19772849 10.1007/BF03262681

[B6] Twetman S Caries prevention with fluoride toothpaste in children: an update Eur Arch Paediatr Dent 2009 10 162 7 19772846 10.1007/BF03262678

[B7] Twetman S Prevention of early childhood caries (ECC)--review of literature published 1998-2007 Eur Arch Paediatr Dent 2008 9 12 8 18328233 10.1007/BF03321590

